# Popliteal block with transfibular approach in ankle arthrodesis: a case series

**DOI:** 10.1186/1752-1947-4-135

**Published:** 2010-05-12

**Authors:** Gabriel A Akra, Alan Middleton, Akinwande O Adedapo, Paul Finn

**Affiliations:** 1 Department of Trauma and Orthopaedics, The James Cook University Hospital, Marton Road, Middlesbrough, TS4 3BW, UK; 2 Department of Statistics, University of Teesside, Middlesbrough, UK

## Abstract

**Introduction:**

Ankle arthrodesis is primarily undertaken to control severe pain in the ankle joint. Immediate postoperative pain is usually treated using oral analgesics, intravenous opiates and regional anaesthesia. The outcomes of ankle fusion, including patient satisfaction studies, are well documented in the literature. However, the advantage of popliteal block in the management of early postoperative pain after ankle fusion for osteoarthritis has not been widely reported. This study aims to determine the role of popliteal block using ankle fusion in the management of ankle osteoarthritis.

**Case presentation:**

Ankle arthrodeses were performed in 27 patients over a five-year period. Eighteen patients were males (one had bilateral arthrodesis) and eight were females. Their mean age was 56 years and they were all Caucasians. The notes and radiographs of the patients were reviewed in retrospect for the duration of their hospital admission, time to union and complications.

**Conclusion:**

Popliteal block is a safe and effective technique for postoperative analgesia in ankle arthrodesis. By using this technique, we achieved a significant reduction in the duration of hospital stay for our patients after ankle arthrodesis. The resultant cost saving was GBP717 for each patient.

## Introduction

Ankle arthrodesis is primarily undertaken to control severe pain in the ankle joint. Immediate postoperative pain is usually treated using oral analgesics, intravenous opiates (Patient Controlled Analgesia) and regional anaesthesia. The outcomes of ankle fusion, patient satisfaction studies and the advantage of regional anaesthesia are well documented in the literature [1], but popliteal block and how it affects the management of these patients has not been widely reported. We carried out a study to review the role of popliteal block in the operative treatment of ankle osteoarthritis using the transfibular approach with multiple cannulated screws to fuse the joint, and to determine the union rate in our practice.

Ankle arthrodesis is the traditional operative treatment for ankle osteoarthritis. It provides the development of a painless, plantigrade and stable foot [2]. There are at least 30 different methods that have been reported to date, including the use of external compression devices combined with open or arthroscopic debridement of joint surfaces, fixation by fibular strut grafts, interposition grafting, and various forms of internal fixation with or without interposition grafting [3,4]. Several authors have reported their series with variable rates of successful primary fusion. Feihel and Uhthoff from Ottawa reported a series with four patients using the Ilizarov technique [5], in whom the fusion rate was 75%. Braly *et al. *in Texas demonstrated a 95% fusion rate in a sample size of 20 patients, using the lateral T-plate technique [6].

The chances of union are higher when the fixation is rigid. Ogilvie-Harris *et al. *demonstrated that three screws are biomechanically better than two screws [7].

The extent of surgical exposure, dissection and debridement makes postoperative pain after ankle arthrodesis quite debilitating. Therefore, surgeons and anaesthetists use various options for postoperative pain control.

## Case presentation

Twenty-seven ankle fusion procedures were performed in 26 patients (one had bilateral ankle osteoarthritis) over a five-year period. There were 18 men and eight women. The mean age was 56 years, with a range of 23 to 83 years. Patient records and radiographs were retrospectively reviewed. The indication for surgery, age, gender, side effects, type of anaesthesia, use of preoperative analgesia, operative technique, duration of hospital stay, need for postoperative analgesia, postoperative follow-up, complications and time to union of the fusion were all recorded.

**Figure 1:**
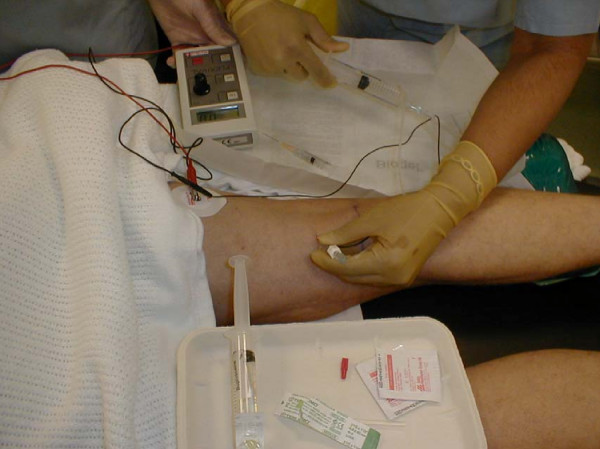
**Popliteal nerve block performed with the patient lying in prone position**.

The patients who received preoperative analgesia had popliteal block. Each patient was laid prone in the anaesthetic room. The electrode of a nerve stimulator was attached to the skin (Figure 1), while the popliteal fossa was aseptically prepared. With the knee flexed and the landmark of a triangle formed by the popliteal skin crease as the base, the medial semimembranosus and lateral biceps femoris were identified. The needle for injecting the anaesthetic was introduced about 7 cm above the skin crease, in the region of the sciatic nerve before its division. The needle was advanced at an angle of 45° until a response registered on the nerve stimulator (Figure 1). Approximately 30 ml of local anaesthetic solution (0.5% bupivacaine) was injected, avoiding injection into the regional blood vessels.

Two surgeons using a single technique performed all the operations.

**Figure 2:**
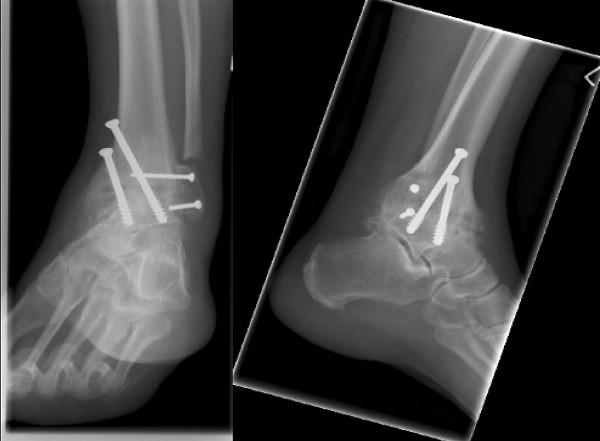
**Anteroposterior and lateral views of a fused ankle**.

At the time of surgery, access to the ankle is gained through a lateral approach centred on the fibula. The fibula is osteotomised approximately 2 cm above the syndesmosis. The distal portion is split in the sagittal plane, taking care to preserve its vascularity. This allows the medial half to be used as bone graft while the lateral half is later fused to the tibia and talus. The approach gives good exposure to the ankle joint and allows adequate preparation of the tibial and talar articular surfaces. The medial malleolus is preserved whenever possible. Curved long spinal curretes are used to clear the medial gutter. Once this is achieved, the surfaces are aligned and held with two parallel guide wires, which are passed distally from the medial side under fluoroscopic control. A bone graft is used to correct defects. The wires are over-drilled, and partially threaded cancellous screws are used to provide compression (Figure 2). The lateral fibular segment, from lateral to medial, is attached to the distal tibia and talus using two parallel screws. The patient's ankle is then immobilised in a back slab and the patient remains non-weight bearing for three to four weeks.

Some patients had popliteal nerve block (Figure 1), while others did not. This was not randomised. All the patients had radiographs at each review outpatient clinic.

There were 26 patients in total, 18 men and eight women, with a male to female ratio of 2:1. The mean age of the patients was 56 years, with a range of 23 to 83 years.

Fifteen patients had popliteal block in addition to general anaesthesia. This helped to manage postoperative pain and shorten the duration of admission. The patients who received popliteal nerve block had a significantly shorter duration of admission (T-test: t(25) = 2.29, p = 0.03) than those who did not. They spent an average of 3.7 days (SD 3.4) admission, which is 3.2 days less than the average duration for those who did not get the block (95% CI: 0.3 to 6.0 days). Two patients had epidural anaesthesia, while one had spinal anaesthesia.

Among the study sample, 96% achieved union. There were no complications recorded from the popliteal block technique. However, we recorded three cases of superficial wound infection, one case of foot and leg swelling, and one case of reflex sympathetic dystrophy. All these were related to the surgery.

## Discussion

Popliteal nerve block is a valuable addition to perioperative care in foot and ankle surgery. It has been found to limit postoperative pain and minimize systemic narcotic complications, as well as maximize patient comfort and promote early discharge [8]. Rongstad *et al. *[9], used 30 ml of 0.5% bupivacaine with epinephrine in the patients they studied. They found that none of their 86 patients had complications related to the block and 95% were satisfied and would have the block again. This procedure, given preoperatively, is safe, augments general anaesthesia, and provides good postoperative pain control in patients with ankle arthrodesis [8,9].

Ankle arthrodesis remains the benchmark of treatment for end-stage arthrosis. Various strategies have been used to reduce the incidence of non-union, including the use of multiple compression screws and larger diameter screws, to improve mechanical stability and compression [10]. A bone graft may be used to enhance the union. Most of the patients in this study had bone grafting at the site of arthrodesis. The graft is sourced from the medial half of the sagitally split osteotomised distal fibula. Besides adding to the factors that enhance bone union, this method avoids the postoperative morbidity that comes with harvesting bone from another site, like the iliac crest. This morbidity, which is often related to pain, may increase the duration of admission for the patient, thereby increasing the cost of ankle fusion.

The cost of health care is a global issue, and one that every health care provider tries to keep at the barest minimum. However, this should not be at the expense of best practice. Luber and Nunley, in their paper on how to reduce the cost of hind foot fusion, noted that the cost savings in a study group of 10 matched pairs of patients who underwent ankle arthrodesis was $9,888, with no increase in complications [1]. The study group had regional anaesthesia and local bone grafting compared to the control group, which had traditional anaesthesia and iliac crest bone graft. These groups are not strictly comparable because the iliac crest graft in the control group may have added to the morbidity and possibly the duration of admission, thus increasing the cost of treatment. On the contrary, we treated both groups in our study equally by ensuring that they all had local bone grafting.

The patients in our study who received popliteal block had a significantly shorter duration of admission. The average duration of admission for this group was approximately four days. However, in the group that did not get popliteal block, the duration of admission was approximately seven days.

The use of opiates and other strong analgesics (oral and parenteral) for postoperative pain control is fraught with complications, such as nausea and vomiting, which tend to be pronounced in the first 24 hours after surgery. These prolong the recovery period for the patient and delay the commencement of postoperative mobilization. The patients who did not receive popliteal block were treated intravenously with morphine for the first postoperative day. This was followed by oral morphine solution which was gradually replaced by co-codamol (a combination of 1 g paracetamol and 60 mg codeine phosphate). We circumvented these with the popliteal block, as all the patients with popliteal block only required mild analgesics like paracetamol for additional postoperative pain control.

The theatre cost of ankle fusion in our centre is £1,400 (including anaesthesia). This is irrespective of whether the patient receives popliteal block in addition to the standard anaesthesia for the procedure or not. However, since the patients receiving popliteal block spent an average of three days less in hospital compared to those without the block, the cost saving was £717 per patient. The cost of hospital admission in our centre is £239 per day.

The reduced length of hospital stay could have been due to other factors like the surgeon's preference for the postoperative management of patients vis-à-vis application of back slab and/or cast, as well as the time to mobilize the patient. However, there was a statistically significant difference between the patients of both surgeons in terms of their duration of admission. Using the analysis of variance (ANOVA) P = 0.001, the surgeon who gave most of his patients popliteal block fared better. Since the determination of time of discharge from hospital depends on the patient's recovery, mobility and pain control, it suggests that the difference between both patient groups is due to the use of popliteal block for pain control rather than the aforementioned factors. The duration of operation did not significantly affect the length of hospital stay: P = 1.9. Neither did age and comorbidities.

In this series, ankle union was achieved in 96% of cases. Certain factors may have contributed substantially to this favourable result. First, the articular surfaces were adequately prepared, and this was helped by the exposure achieved using the transfibular approach. We preserved the lateral half of the sagitally split distal fibula with soft tissue attachment. This was used as a lateral support to the fusion. An added advantage of this approach is that with the lamina spreader, an excellent exposure is achieved for adequate resection of the articular cartilage and minimum subchondral bone, thus preserving the limb length.

Second, maximum contact and compression of viable cancelous bone ends was achieved using multiple cannulated screws. Rigid fixation, compression and adequate biologic environment are known to be the key elements for bone healing [5,11-13]. Alonso-Vazquez *et al. *[14] suggested that there is better stability with three-screw arthrodesis than two-screw arthrodesis in their finite element analysis. There may be concerns about compromising the biologic environment that is required for the union of bone, particularly when an increased amount of hardware is used in a relatively small bone. Brodsky *et al.*, in their analysis of talar surface area occupied by screw fixation in ankle fusion, found that the maximal surface area of talus occupied by screws occurred when using three 7.3 mm screws [10]. This configuration used 16% of the possible talar surface area available for arthrodesis. They concluded that the use of additional screw fixation when performing an ankle arthrodesis does not sacrifice a major amount of the tibiotalar contact area and will most likely not affect the biologic environment needed to obtain fusion. In general, a more secure fixation is associated with increased rates of fusion.

## Conclusion

Popliteal block is a safe and effective technique for postoperative analgesia in ankle arthrodesis. By using this technique, we achieved a significant reduction in the duration of hospital stay for our patients.

## Abbreviations

ANOVA: analysis of variance; CI: confidence interval.

## Consent

Written informed consent was obtained from the patients for publication of this case report and any accompanying images. Copies of the written consent are available for review by the Editor-in-Chief of this journal.

## Competing interests

The authors declare that they have no competing interests.

## Authors' contributions

GA collated and analysed the data, wrote the abstract, introduction, and discussion, and did the literature search. AM wrote the case presentation and served as the assistant surgeon. AO performed patient selection and most of the surgical operations. He also contributed the figures used in this manuscript. PF was responsible for the statistical analysis. All authors read and approved the final manuscript.
